# Predictive factors for complexity in abdominal wall hernias: a literature scope review

**DOI:** 10.1590/0100-6991e-20243670-en

**Published:** 2024-04-17

**Authors:** CIRÊNIO DE ALMEIDA BARBOSA, MATHEUS HENRIQUES SOARES DE FARIA, BRUNO AMANTINI MESSIAS

**Affiliations:** 1 - UFOP (Universidade Federal de Ouro Preto), Departamento de Cirurgia, Ginecologia, Obstetrícia e Propedêutica da Escola de Medicina. - Ouro Preto - MG - Brasil; 2 - UFOP (Universidade Federal de Ouro Preto), Escola de Medicina - Ouro Preto - MG - Brasil; 3 - Hospital Geral de Carapicuíba, Departamento de Cirurgia - Carapicuíba - SP - Brasil

**Keywords:** Abdominal Hernia, Hernia, Classification, Incisional Hernia, Hérnia Abdominal, Hérnia, Classificação, Hérnia Incisional

## Abstract

**Introduction:**

Abdominal wall hernias encompass both ventral and incisional hernias, often poorly classified regarding complexity in general. This study aims to conduct a review on the primary topics related to defining the complexity of ventral hernias.

**Methods:**

this is a scope review conducted following the guidelines recommended by the PRISMA-ScR directive. Searches were carried out in electronic databases including PubMed, LILACS, and EMBASE, using the descriptors: Abdominal Hernia, Hernia, Ventral Hernia, Incisional Hernia, Complex, Classification, Classify, Grade, Scale, and Definition. Combinations of these terms were employed when appropriate. Inclusion criteria encompassed articles with definitions and classifications of complex hernias, as well as those utilizing these classifications to guide treatments and patient allocation. Synonyms and related topics were also considered. Articles outside the scope or lacking the themes in their title or abstract were excluded. The database search was conducted up to July 29, 2023.

**Results:**

several hernia classifications were identified as useful in predicting complexity. For this study, we considered six main criteria: size and location, loss of domain, use of abdominal wall relaxation techniques, characteristics of imaging exams, status of the subcutaneous cellular tissue, and likelihood of recurrence.

**Conclusion:**

complex abdominal wall hernias can be defined by characteristics analyzed collectively, relating to the patients previous clinical status, size and location of the hernia defect, status of subcutaneous cellular tissue, myofascial release techniques, and other complicating factors.

## RESUMO

**Introdução:**

As hérnias da parede abdominal, englobam em seu conceito as hérnias ventrais e incisionais. Essas hérnias são mal classificadas quanto à complexidade de forma geral. Esse estudo tem por objetivo realizar uma revisão sobre os principais tópicos relacionados à definição de complexidade das hérnias ventrais.

**Métodos:**

trata-se de revisão de escopo realizada com as orientações preconizadas pela diretriz PRISMA-ScR. Foram realizadas buscas nas bases de dados eletrônicas do PubMed, LILACS e EMBASE, empregando os descritores: “Abdominal Hernia”; Hernia; “Ventral Hernia”; “Incisional Hernia”; Complex; Classification, Classify, Grade, Scale e definition. Combinações entre os termos foram utilizadas quando oportunas. Os critérios de inclusão abrangiam artigos com definições e classificações de hérnias complexas, além daqueles que usavam essas classificações para orientar tratamentos e alocação de pacientes. Sinônimos e tópicos relacionados também foram considerados. Artigos fora do escopo ou sem os temas no título ou resumo foram excluídos. A busca nas bases de dados foi realizada até o dia 29/07/2023.

**Resultados:**

diversas classificações de hérnias foram identificadas como sendo úteis para prever complexidade. Consideramos para esse estudo seis critérios principais: tamanho e localização, perda de domicílio, uso de técnicas de relaxamento da parede abdominal, características dos exames de imagem, status do tecido celular subcutâneo e chance de recorrência.

**Conclusão:**

as hérnias complexas da parede abdominal podem ser definidas por características analisadas conjuntamente e que estão relacionadas ao estado clínico prévio do paciente, tamanho e localização do defeito herniário, status do tecido celular subcutâneo, técnicas de liberação miofascial e outros fatores complicadores..

## INTRODUCTION

Most ventral hernias (VH) classified as complex come from patients with incisional hernias (IH). IH constitute a major public health problem and cause significant morbidity and mortality. They have an estimated prevalence of up to 5% in the general population and may be present in 4% to 15% of patients undergoing laparotomy^1^ ^,^ ^2^. In addition to high morbidity and impaired quality of life, the costs of surgical procedures, hospitalizations, and treatments are high and increasingly occupy a portion of public and private health expenses.

IH occurs due to weakening of the abdominal wall caused by previous surgical incisions. However, primary hernias of the abdominal wall, which are those that occur due to “natural” defects (eg, umbilical hernias with loss of domain), can also be classified as complex. Primary hernias have a different etiopathogenesis compared with abdominal wall IH, the former being those that occur in places without a previous incision, whose pathogenesis is related to the anatomy of the abdominal wall and points of weakness in a fascial plane without sufficient musculature to sustain elevations in intra-abdominal pressure. The most common primary hernias are umbilical and epigastric hernias, mainly in the midline^3^.

The classification of complex hernias still lacks consensus in the literature and the various predictive criteria available lead to heterogeneity of knowledge. The central purpose of any classification system lies in improving comparability between various studies and their results and is therefore valuable for establishing evidence on how professionals should approach specific situations. Contemporary times have offered us multiple classificatory approaches and patients stratification criteria, which is why it is necessary to formulate a single system. The secondary purpose of a classification system would be to consolidate the results and formulate therapeutic guidelines based on evidence, using it as a basis. When a classification becomes widely accepted and shows demonstrable ability to improve patients’ outcomes by grouping them according to the system it proposes, one can use it in future studies to more clearly appraise the outcomes achieved. Each of the different classifications considers factors that are not unanimous, many of which are repeated exhaustively.

The main objective of this article is to present the most well-known classifications used in medical practice with the aim of understanding the main characteristics that must be considered to classify a hernia as complex, whether based on morphological or clinical aspects of the patients or on imaging studies.

## METHODS

We carried out this study by searching the electronic databases of PubMed, LILACS, and EMBASE, using the following terms: “Abdominal Hernia”; “Hernia”; “Ventral Hernia”; “Incisional Hernia”; “Complex”; “Classification”, “Classify”, “Grade”, “Scale”, and “definition”, using combinations between terms with the Boolean operators AND and OR. We summarize the research strategy in Table 1. The inclusion criteria were articles that had in their title or abstract the definition of complex hernias and classification for complex hernias, as well as articles that used the classifications to allocate patients to studies and to select appropriate treatments. We also included other topics and synonyms that explained and classified complex hernias. We excluded articles that did not address the research topics in their title or abstract. Among the eligibility criteria, we also considered that redundant, old classifications that were presented again by more up-to-date articles were only cited among the references for each topic. We did not assess risk of bias, as we were interested in complex hernia definitions and not in strict methodological quality. We carried out the search in the databases until 07/29/2023.

**Table 1:** Methodology used to search databases..

Terms used	Number of results
#1 | ABDOMINAL HERNIA #2 | ABDOMINAL WALL RECONSTRUCTION #3 | VENTRAL HERNIA #4 | INCISIONAL HERNIA #5 | COMPLEX #6 | CLASSIFICATIONS #7 | CLASSIFY #8 | SCALE #9 | GRADE #10 | DEFINITION	
(#1 OR #2 OR #3 OR #4) AND #5 AND (#6 OR #7 OR #8 OR #9 OR #10)	181
(#1 OR #2 OR #3 OR #4) AND #10	201
(#1 OR #11 OR #3 OR #4) AND #6	304
#1 AND #5 AND #6	11
#2 AND #5 AND #6	54
#3 AND #5 AND #6	44
#4 AND #5 AND #6	25

The search produced 820 bibliographic references, which in the end produced 530 non-duplicate references. Two reviewers who worked independently read and analyzed the non-duplicate articles, using the EndNote™ application to organize references and read abstracts. Disagreements related to the selection of articles for review were resolved through discussion between the reviewers when necessary. The selection of non-duplicated works yielded 53 manuscripts that contained in their title or abstract the definition of complex hernias or classification systems for hernias with complexity factors. Two reviewers read these manuscripts in full. Of the works read in full, we selected 26 to produce this review. We also scrutinized the reference list of articles selected for the review, with the purpose of identifying potentially eligible studies that were not found in database searches. This search yielded 13 more articles. In the end, we considered 39 articles to produce this scoping review.

## RESULTS

Due to the lack of uniformity in the classifications of complex abdominal wall hernias, we believe that stratifying them into topics would facilitate the understanding of the main characteristics to be analyzed in complex cases. We present the results obtained from exploring the classifications of complex hernias in this review in six topics separated by central criteria used by each article to classify the complexity and risk of unfavorable outcomes. The classifications found evaluate abdominal hernias in relation to size and location, loss of domain, need to use abdominal wall relaxation techniques, characteristics of imaging exams, status of subcutaneous cellular tissue, and chance of recurrence. All these parameters were recognized as increasing the likelihood of difficulty during the repair of abdominal wall hernias, with the complexity in most studies being directly related to the number of adverse events and negative outcomes during and after surgery. Within each of the topics presented, we respected chronological order to better understand each criterion. The work from Slatter et al. for defining complexity criteria does not address a specific criterion that must be taken into consideration, so we present the results of their study separately from the other six topics.

This way, the parameters analyzed with the classifications selected in this review improve stratification and individualize the circumstances of patients with abdominal hernias and can help reduce the risk of negative outcomes through better predictability when faced with a patient with suspected complex abdominal hernia. 

### Size and location

The first classifications used to classify hernias as complex were based almost solely on their location and size. The most established classifications on this topic are exemplified below.

### Chevrel and Rath classification (2000)

The objective of these authors was to propose a classification whose central value was simplicity in stratification and predictability of results. This classification was divided into four parameters: location of the incisional hernia, width of the hernia orifice, number of recurrences, and treatment results.

This classification was validated based on a retrospective study carried out between 1980 and 19984, with 435 patients with abdominal IH. Three initial parameters were used to stratify patients - location, size, and recurrences (SWR classification). This study found that the width of the hernia would be the most important parameter in relation to complexity and complications.

Therefore, size and recurrence were considered determinant for outcomes, and were thus used for the proposed classification. (1A/B; 2A/B; 3A/B; 4A/B) (Table 2).

**Table 2:** Chevrel and Rath SWR Classification. W: size; A: recurrence; B: recurrence + number of recurrences.

Location	Type
M (Midline)	1: A or B
(w <5cm)	
M (Midline)	2: A or B
(w: 5-10cm)	
L (Lateral)	3: A or B
(w: 10-15cm)	
L (Lateral)	4: A or B
(w >15cm)	

### Classification by Muysoms et al. (EHS) (2009)

This classification was carried out with the aim of stratifying primary abdominal wall hernias and a subgroup of incisional hernias^5^. They identified that the location of the primary hernia and the size of the defect as decisive factors for the result and should be present when formulating the classifications. Primary hernias were divided into median (epigastric and umbilical) and lateral (Spiegel and lumbar). The size was defined by the diameter, and the stratification occurred in three groups, with cutoff values of 2cm and 4cm (Table 3).

**Table 3:** Table for classifying primary abdominal wall hernias, according to Muysoms et al.

EHS Classification of primary abdominal wall hernias	Small <2cm	Average ≥2-4cm	Big ≥4cm
Midline | Epigastric			
Midline | Umbilical			
Lateral | Spiegel			
Lateral| Lumbar			

**Figure 1:**
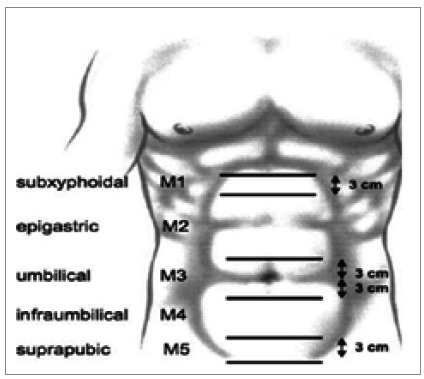
Localization classification of median incisional hernias of the abdominal wall described by Muysoms et al. M1: subxiphoid (from the xiphoid to 3cm caudally); M2: epigastric (from 3cm below the xiphoid to 3cm above the umbilicus); M3: umbilical (from 3cm above to 3cm below the umbilicus); M4: infraumbilical (from 3cm below the umbilicus to 3cm above the pubis); M5: suprapubic (from the pubic bone to 3cm cranially).

**Figure 2:**
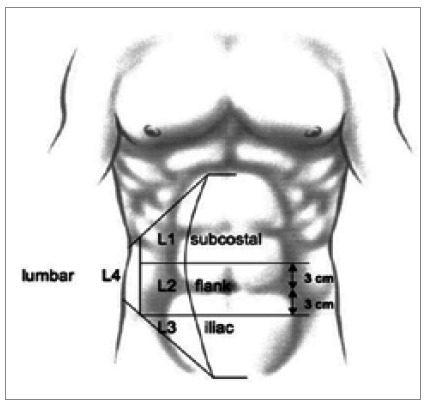
Localization classification of lateral incisional hernias of the abdominal wall described by Muysoms et al. L1: subcostal (between the costal margin and a horizontal line 3cm above the umbilicus); L2: flank (lateral to the rectal sheath in the area 3cm above and below the umbilicus); L3: iliac (between a horizontal line 3cm below the umbilicus and the inguinal region); L4: lumbar (lateral-dorsal, past the anterior axillary line).

For incisional hernias, there was also a consensus that the location and size of the defect would be essential for classification. Other variables were not included. For location, it was proposed to group the hernias that did not cross the semilunar line of the rectus abdominis muscle as median and those that were external to this limit as lateral. Subdivisions for medial and lateral incisional hernias have been defined and are detailed in Figures 1 and 2.

The classification of hernias that extend into several zones (M) of the abdominal wall was not reached by consensus. One suggested approach was to assign the most challenging or representative M zone for the hernia. The prioritized M zones are subxiphoid (M1) and suprapubic (M5), followed by umbilical (M3) and, finally, epigastric (M2) and infraumbilical (M4). Therefore, a hernia extending from M1 over M2 to M3 would be classified as M1 (Table 4).

**Table 4:** Table for classifying incisional hernias of the abdominal wall, according to Muysoms et al.

Median	SUBXIPHOID - M1
Median	EPIGASTRIC - M2
Median	UMBILICAL - M3
Median	INFRAUMBILICAL - M4
Median	SUPRAPUBIC - M5
Lateral	SUBCOSTAL - L1
Lateral	FLANK - L2
Lateral	ILIAC - L3
Lateral	LUMBAR - L4
HERNIA RECURRENCE?	YES ( ) / NO ( )
Length: (cm)	Width: (cm)
WIDTH	W1: < 4cm ( ) W2: ≥4- 10cm ( ) W3: ≥ 10cm ( )

The definition of the size of the hernial defect used two components, namely width and length, considering that IH defects can be multiple and poorly localized.

### Loss of domain

During the repair of abdominal wall hernias, surgeons may encounter chronic extrusion of viscera from the abdominal cavity, resulting in a decrease in myofascial elasticity, muscle retraction or atrophy, and a chronic reduction in the volume of the intra-abdominal cavity^6^.

A current systematic review reports that loss of domain is a term that was found in the literature with disparate definitions or completely omitted in IH studies^7^. However, a definition produced by consensus of surgeons specialized in the abdominal wall characterized loss of domain as hernias that present irreducibility due to lack of intra-abdominal space, need to use reconstructive techniques to facilitate reduction, and present an increased risk of complications due to increased intra-abdominal pressure^8^.

Volumetric methods are a way to predict loss of domain more accurately. The classification by Tanaka et al., which uses tomographic exams to evaluate loss of domain, is one of the most used.

This condition is one of the major complexity criteria during the repair of ventral hernias and the attempt to reduce the contents without preparing the abdominal cavity can cause respiratory and circulatory disorders due to the sudden increase in pressure, called abdominal compartment syndrome^9^ ^-^ ^11^, which can lead to worse outcomes.

We present the classification by Tanaka et al., which was mainly used as a criterion for the predictability of loss of domain: 

### Classification by Tanaka et al. (2010)

**Figure 3:**
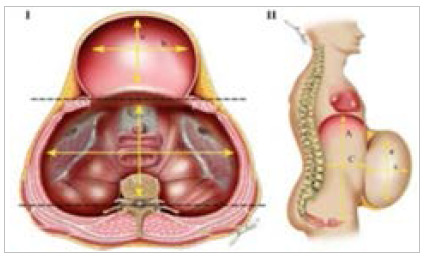
Axial (I) and sagittal (II) measurements taken during volumetry of the abdominal cavity and hernial sac. A, B, and C are measurements of the abdominal cavity, and a, b, and c are measurements of the hernia cavity.

The use of physical examination as the only element in predicting the contents of the hernial sac is of little value, leading to frequent errors due to the thickness of the abdominal wall and confounding factors during prediction, such as in cases of obesity. Imaging exams are important tools in the predictability and conduction of surgical management of large ventral hernias. The classification by Tanaka et al.^12^ uses computed tomography to stratify patients, using a ratio between hernia volume (HV) and abdominal cavity volume (ACV). During cavity measurement, both cavities are considered as ellipsoids, allowing estimation of their volumes. The values are acquired by measuring the longitudinal (A,a), transversal (B,b) and anteroposterior (C,c) diameters of each cavity, using the largest measurement of all sections, even if these measurements are obtained from different cuts. For these calculations, it is essential to have reference points that function as delimiters. In this context, the anterior limit of the abdominal cavity is established through a line that connects the muscle groups of the healthy wall, while the posterior limit is defined by a line that crosses the transverse process of the vertebrae. The cranial limit of the abdominal cavity, for the cranio-caudal measurement, corresponds to the first axial cut that shows the diaphragm, while the inferior or caudal limit of the abdominal cavity corresponds to the last axial cut that shows the coccyx. The lateral limits are determined by the parietal peritoneum on each side. Regarding the measurement of the hernia sac, its limits include the parietal peritoneum of the hernia sac at the upper, lower, and lateral ends of the hernia sac. The only exception is the posterior limit of the hernial sac, which is established by the same line that delimits the anterior limit of the abdominal cavity, that is, the line that unites the muscle groups of the healthy wall. The equation that simplifies the ellipsoid volumes is HV (or ACV) ≈ 0.52×a×b×c (Figure 3).

Obtaining a ratio between the contents of the hernia sac and abdominal contents greater than or equal to 25% classifies the patient’s hernia contents as losing their domain. In these cases it may be necessary a preoperative progressive pneumoperitoneum program, botox and/or other adjuvant techniques.

Despite the ease of performing calculations using this technique, the criteria selected to define which patients should undergo a preoperative pneumoperitoneum scheme were defined quite arbitrarily^12^ ^,^ ^13^. However, later work by Al-Mansour et al. found a strong correlation between simple mathematical formulas, as described by Tanaka et al., and the surface area and volume measurements obtained through 3D Computed Tomography reconstruction^14^.

### Regarding the analysis of factors predicting the need for a tension-free relaxation and closure technique

To achieve tension-free closing, it may be necessary to use additional techniques, such as the component separation technique, with the aim of strategically dividing the myofascial layers of the abdominal wall and, consequently, relieving tension in the fascial approximation^15^ ^-^ ^17^. Due to the high risk of closure under tension in complex IH, which can increase the risk of compartment syndrome, criteria have been established to identify the need for techniques that promote closure without tension^18^ ^,^ ^19^. The use of the size of the ventral hernia as an isolated component for the selection of patients who require relaxation techniques proved to be unreliable because of individual characteristics of each patient reflecting on the compliance of the abdominal wall^14^ ^,^ ^20^ and the fact that fascial defects of small proportions may be correlated with large hernia sacs and loss of domain^12^ ^,^ ^21^.

Therefore, appropriate, scientifically validated criteria were adopted to predict the need to use relaxation techniques during the repair of ventral hernias, with the main classifications being exemplified below

### Classification by Ammaturo and Bassi (2005)

This classification added another parameter to the Chevrel and Rath^22^ classification. This parameter was the ratio between the surface of the anterior abdominal wall (SAAW), using the bi-iliac and xiphopubic lines as reference, and the surface of the hernial defect (SHD), a low such ratio being a predictor of high tension of the abdominal wall when performing primary closure of the defect and greater tendency to compartment syndrome.

To evaluate this criterion, SAAW is divided by SHD and values <15 are predictors of tension during closure using only the Rives technique. The authors proposed this cutoff line based on their own experiences and report the use of intraperitoneal mesh to correct defects prone to tension. This measurement was an incipient concept of what was to come in the classification criteria predicting the need for tension-free relaxation and closure techniques.

### Sabbagh Classification (2011)

**Figure 4:**
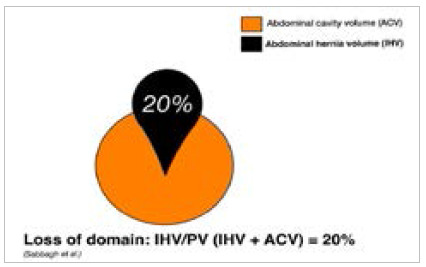
Analysis of loss of domain using Sabbagh’s et al. volumetric method. Image by authors.

Repair of major hernias with loss of domain may require devices and surgical techniques to avoid compartment syndrome. The Sabbagh classification allows the surgeon to predict when fascial closure will occur without tension. During the study, variables were postulated that the researchers empirically considered as relevant for the closure of the fascia without tension, namely age, patient’s body mass index, the width, length, and surface area of the IH, and the ratio between the IH volume and the peritoneal volume (IHV/PV). To calculate the volumes of IH and peritoneum, the authors used computed tomography scans and specialized software, a limiting factor for routine application^13^ ^,^ ^23^. During the study, they found that an IHV/PV ratio of less than 20% is a significant predictive factor for a simple abdominal closure technique (Figure 4). In cases where the IHV/PV measurement is greater than or equal to 20%, the surgeon must prepare the intervention differently, using additional relaxation techniques to reduce tension on the suture line. The use of fascioplasty and even surgical flaps should be considered in such cases^24^ ^,^ ^25^.

The small study sample, which included only 17 patients, and the need for specialized software to perform the measurement were limiting factors for the routine application of the method^13^.

### Classification of Christy ‘s et al. (2012)

**Figure 5:**
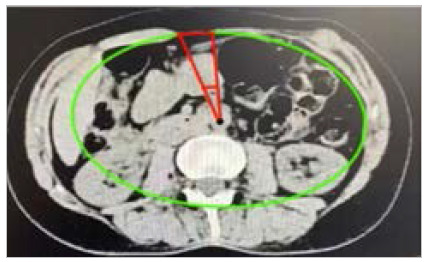
Calculation of the component separation index using the reference points proposed by Christy et al.

In 2012, Christy et al.^21^ published the results of a study carried out with 36 patients demonstrating a new index that could be used to predict the need of a repair with interpositional mesh and the separation of components to achieve adequate midline closure. The Component Separation Index (CSI) is a value derived from preoperative CT scans and offers an alternative approach to simply assessing the distance between the edges of the hernia or the total area of the defect. This index is constructed from data obtained from abdominal computed tomography, including the angle of diastasis of the rectus abdominis muscles in relation to the aorta. The calculation of the index involves dividing the diastasis angle (AD) by 360, and the center of the diastasis angle is measured using the abdominal aorta as a reference (Figure 5).

[Fig f6]

**Figure 6 f6:**
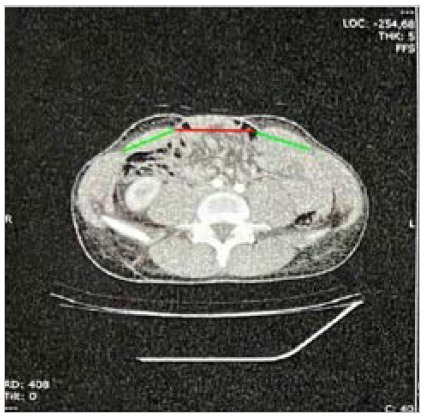
Calculation of the Love index, ratio between the widths of the rectus abdominis muscles and of the defect. A: right rectus abdominis; B: left rectus abdominis; and C: hernia defect. Axial tomographic image of a midline incisional hernia after complicated appendectomy and peritoneostomy.

This figure establishes a correlation between the size of the defect and the unique biometric profile of each patient^18^ ^,^ ^21^, which allows for more meaningful comparisons. As the component separation index approaches 0.21, the probability of opting for an interpositional mesh repair increases considerably, in addition to the need to use techniques of components separation.

### Classification by Love et al. (2021)

Before the studies by Love et al.^26^, Carbonell et al.^17^ reported, in a very similar way, an objective parameter that would be able to predict when primary closure of the defect would not be feasible. When the maximum width of the defect approaches or exceeds twice the width of the rectus abdominis muscle, direct closure of the defect would not be possible. This method has one of the simplest calculations, but it lacks scientific validation.

Later, in a retrospective study of 342 patients, Love et al. examined CT imaging data, measuring the width of the hernia, the width of the right and left rectus muscle, and the angle of the defect. From these data, they performed calculations to determine the relationship between the width of the rectus muscles and the width of the defect. The ratio between these widths was obtained through the simple sum of the widths of the right and left rectus muscles, divided by the width of the hernia. Thus, the researchers found that the rectum width/defect width ratio is a practical and reliable tool to predict the ability to close the hernial defect during the Rives-Stoppa procedure, without the need for additional techniques. Their statistical analysis showed that, when the ratio was greater than two, patients did not need other relaxation techniques in 90% of cases.

The study also assessed the validity of the component separation index, which was calculated following the method described by Christy et al.^21^. The results obtained demonstrate that the component separation index was predictive of the need for myofascial release when greater than 0.15 in 76.3% of cases and that the average component separation index of patients in whom closure without relaxation was not possible was 0.218. These values were very close to those in the original study.

### Imaging exams

The quantitative and qualitative measurements presented in the imaging exams are of great value for the preoperative prediction of component separation and the use of adjuvant techniques during IH repair. The size of the hernia defect, the muscular quality of the abdominal wall, and the volume of the hernia can be evaluated with good results using computed tomography^20^. Classification by imaging exams presupposes a study using standardized tomographic reports, containing all the information for predicting complexity.

Current studies use imaging exams to refine the analysis of predictive factors, and many of them use tomographic analyzes in their classifications^20^ ^,^ ^27^.

### Classification by Gandhi et al. (2023)

Gandhi et al.^20^ used the growing space of computed tomography scans in the current approach to ventral hernias to prepare a standard report, with the intention of bringing together the main characteristics necessary for the classification and management of these patients. The description included the following information:

1. location and size of the hernia defect, in accordance with the recommendations of the European Hernia Society^5^;
2. volume of the hernial sac and the abdominopelvic cavity, also considering the Tanaka index for loss of domain^12^;
3. measurement of the component separation index from Christy et al.^21^;
4. Application of the Carbonell index^17^, with refinements later described by Love et al.^26^;
5. description of the contents of the hernia sac, including mobility and possible characteristics observed during the Valsalva maneuver;
6. presence of fistulas, obstruction/strangulation, previously implanted mesh, and separation of components performed in another surgery; and
7. quality, thickness, and symmetry of the abdominal muscles (in some cases the sarcopenia index must be used)^6^ ^,^ ^28^.

The information contained when formulating the standard tomographic report from Gandhi et al. help the surgeon in evaluating the complexity of the hernial defect, also preparing the main approaches necessary to achieve the best outcomes.

### Status of the subcutaneous cellular tissue

The status of the subcutaneous cellular tissue is a great predictor of the complexity during hernia repair^10^ ^,^ ^11^. Many studies address specific characteristics of the patient’s clinical condition to predict the best technique to be used, surgical site infection being an extremely important one^29^ ^,^ ^30^. Studies have reported infection rates after ventral hernia repair ranging from 4% to 16%. Other clean surgical procedures have a much lower infection rate (<2%)^31^. Regardless of the technique used, the results may depend significantly on the appropriate management of the skin and subcutaneous tissue^30^. In fact, complications such as wound dehiscence and surgical site infections can pose a serious threat to the reconstruction of the abdominal wall, especially when the synthetic mesh is exposed and vulnerable to infection, thus establishing a vicious cycle. The presence of fistulas, infections, and the patient’s clinical comorbidities are aggravating factors when predicting complexity10, so one should include the main classifications related to contamination, healing, and tissue status.

### Classification of the Ventral Hernia Working Group (VHWG) (2010)

In September 2008, the VHWG^31^ held an expert meeting with the objective of promoting a hernia classification system that relates patient and wound risk factors to the risks of occurrences at surgical sites (OSS), especially infection. OSSs that are common after ventral hernia repair include infection, seroma, wound dehiscence, and fistula formation. When there is an increased risk for OSS based on assessment of risk factors, surgeons may utilize supplemental techniques, such as the use of biological repair in place of synthetic mesh.

The VWHG classification system demonstrates a model for assessing the risk of infection at the surgical site based on the individual characteristics of patients and, mainly, their clinical factors prior to the surgical procedure. Each degree presented in this classification is associated with the presence or absence of risk factors, not rigidly categorizing the patient into a specific group, but indicating a continuous spectrum of risk. This classification does not consider the size or complexity of the hernia defect independently, and a patient with a relatively small hernia but with clear signs of infection would still be considered grade 4. Thus, we exemplify the stratification carried out in the study, which divided patients into four degrees regarding the probability of OSS:

1. Grade 1: young and healthy individuals, with no signs of comorbidities.
2. Grade 2: patients with comorbidities that increase the risk of surgical site infection, without evidence of wound contamination or active infection. The comorbidities found during the literature review of the study include COPD, smoking, nutritional status, immunosuppression, obesity, low serum albumin, coronary artery disease, and use of corticosteroids. Furthermore, the following thresholds have been proposed for an independent increase in the risk of infection: blood glucose above 110mg/ dL (hemoglobin A1c >7.0) and age above 75 years.
3. Grade 3: patients with probable wound contamination, being a higher risk category. Factors that suggest contamination include the presence of a nearby stoma, violation of the gastrointestinal tract, or history of wound infection.
4. Grade 4: Infected patients who are at greater risk of occurrence at the surgical site. Grade 4 features include active infection, especially infected synthetic mesh, and septic dehiscence.

This classification stratifies clinical and wound factors that directly influence the status of regional cellular tissue, so patients classified with higher degrees require adjuvant techniques during surgery, with the use of biological repair and treatment of chronic infections, being predisposed to greater complexity and worst outcomes.

### Classification of the Ventral Hernia Working Group modified by Kanters et al. (2012)

The classification proposed by the VWHG in 2010 was based on the best available evidence but did not obtain verification and validation through clinical use and studies^31^. Kanters et al.^30^ proposed modifying the VHWG classification based on a population study carried out prospectively with 299 patients with IH from Case Medical Center. This study grouped patients according to their degree of OSS risk using the classification proposed by the VHWG, later analyzing infection incidence. The study would attest to the usefulness of this classification in predicting OSS, especially infection.

The authors observed that grade 3 patients with clean wounds and only a history of previous wound infection presented an incidence that was statistically comparable to that of grade 2 patients. On the other hand, patients categorized as having grade 3 hernias for other reasons, such as cases of probable contamination, did not show statistical differences from grade 4 patients.

Therefore, the authors proposed that the classification be modified to include patients with a history of previous wound infection as grade 2 patients and combine the remainder of grade 3 and grade 4 patients, promoting a new classification that would demonstrate significant differences between each grade independently, rendering the classification more precise (Table 5).

**Table 5:** VHWG classification modified by Kanters et al. The probability of OSS was determined for each group based on univariate analysis.

DEGREE	Description	Occurrence in Surgical Site
GRADE 1	Low risk of complications; No history of wound infection	14%
GRADE 2	Smoker; Obese; COPD; DM; History of wound infection	27%
GRADE 3	Clean-contaminated; Contaminated; Dirty	46%

### Risk of recurrence

Recurrence is one of the most feared outcomes after hernia surgeries, being considered a predictor of complications and complexity in future surgeries. Despite significant advances in IH repair techniques and technologies, recurrence rates remain high. Previous mesh infections, recurrent repairs, and surgical site infection significantly increase the risk of recurrence^32^. The ability to predict recurrence risk assists the surgeon during surgical decision-making and guides surgery preparation.

Chevrel and Rath, in their pioneering classification, analyzed the recurrence rates of their patients and used it as part of the SWR classification^4^. Ammaturo and Bassi et al. proposed two main parameters to explain the high recurrence rates in IH corrections: surgical site infection and excessive tension in the midline^22^.

More recent studies have raised the hypothesis of what would be the most important complication to be analyzed (recurrence or surgical site infection). Several conditions predispose and increase the chances of recurrence, with patients with postoperative infections having recurrence rates that can reach 80%. Other data that influenced the risk of recurrence were patients’ clinical factors and the surgical technique used^31^ ^,^ ^33^.

### VHWG classification modified for recurrence rates by Hodgkinson et al. (2021)

This was the first study to demonstrate that the VHWG classification system modified by Kanters et al. is a valid tool for stratifying patients according to the chances of recurrence and clearly showed that surgical infection is the factor most responsible for recurrences^33^ ^,^ ^34^. Statistical graphing with a 15-year follow-up predicted the probability of new hernia surgery in 11% of patients classified as grade 1, 14% in grade 2, and 20% in grade 3. Most recurrences occurred in the first 3 years after the initial surgery, indicating 6% for grade 1, 8% for grade 2, and 12% for grade 3.

When assessing recurrence risk, the most important factors were the same as those addressed in previous literature, adding only inflammatory bowel disease as an associated risk factor. Thay demonstrated, therefore, that the VWHG classification modified by Kanters et al. had good results in predicting the need for additional surgery, building an appropriate stratification method. 

### Classification by Slater et al. (2014)

Slater et al.^10^ addressed some clinical criteria of the patients and descriptive criteria of the hernial defect that directly influence management complexity. Although it does not encompass all the characteristics that indicate the degree of complexity in the management of IH^14^ ^,^ ^20^ ^,^ ^35^, the analysis jointly addresses essential factors that must always be taken into consideration. Four components were defined: size and location, soft tissue contamination/condition, patient history/risk factors, and clinical scenario.

Regarding size, as well as the classification by Chevrel and Rath^4^, they advocate the width over the length of the defect and consider that the surface can be misinterpreted most of the time. Defects larger than 10cm have been empirically proposed as a cutoff for complexity if there are other complicating factors^10^. Regarding location, lumbar, lateral, and subcostal hernias were considered complex due to the difficulty in fixing the mesh and the risk of recurrence.

The soft tissue contamination component uses four wound criteria as a general classification: clean (I), clean-contaminated (II), contaminated (III), and dirty/infected (IV). Hernias of grade III (contaminated) and IV (dirty/infected) are considered complex. The condition of tissues during surgical repair is important and can be influenced by tissue loss, previous grafting, hypertrophic scars, extensive debridement, muscle denervation, ulcers, and other related conditions^32^ ^,^ ^36^ ^,^ ^37^.

A recurrent hernia is considered a risk factor for a new recurrence, given the pathogenesis of these injuries, which often involve individual issues^32^ ^,^ ^38^. Furthermore, correction surgery makes the quality of the tissues in the region less adequate, this happening because tissue dissection, mesh removal, fascial retraction, and debridement are factors that make the tissues more vulnerable and less adapted to surgical repair^10^ ^,^ ^32^ ^,^ ^39^.

Risk factors reported during the study were age, male sex, chronic lung disease, cough, ascites, jaundice, anemia, emergency surgery, wound infection, obesity, steroid use, hypoalbuminemia, hypertension, perioperative shock, and type of surgery^10^. Furthermore, multiple hernia defects, unsatisfactory healing, impossibility of primary closure, and fistulas are also considered complex. Hernia reducibility and the presence of obstruction in its classification were considered.

Finally, patients were grouped according to the four presented criteria and subsequently into three classifications, minor, moderate, and major, depending on the number of factors that influence post-surgical healing and convalescence.

## DISCUSSION

For a comprehensive approach with adequate scientific evidence during the evaluation and management of abdominal wall hernias, it is recommended to use different descriptions and classifications recognized in the medical literature, and the use of multiple parameters can contribute to individualizing the cases. Each of the classifications was developed with specific motivations, but many are interconnected. The classification from Sabbagh et al., whose main objective was to describe a method for predicting the separation of components, was also used to predict loss of domain, since it is a volumetric calculation. Despite the classification of Gandhi et al. used a specific tomographic report as the core of their stratification to classify each case, many of the classifications with different central proposals have in common the use of imaging exams.

The classification from Slatter et al is extremely comprehensive, bringing together factors related to clinical parameters, as in the manuscripts by Kanters et al. and Hodgkinson et al., and descriptive parameters of the hernia defect, such as the classifications by Muysoms et al.

To accurately describe the multitude of factors that can influence the complexity of abdominal hernia repairs, it is advantageous to stratify each of the parameters analyzed, as in the topics of this review. Therefore, to describe the location and size of the hernial defect, it is recommended to use the EHS description, as presented by Muysson et al. This is a valid approach, considering that the classification is updated and widely disseminated, in addition to being supported by expert consensus carried out by the EHS council. The classification by Chevrel et al. is simplistic and predictable, applying factors that could contribute to the practical daily classification of abdominal hernias. However, the SWR method must be used with caution, as it is a retrospective study carried out more than twenty years ago and which defined the classification groups quite arbitrarily. Furthermore, the classification by Chevrel et al. cannot characterize some hernia defects precisely, such as multiple defects, which may have their description compromised.

The prediction of loss of domain can be based on the description by Tanaka et al. and Sabbagh et al. The study by Tanaka et al. had an arbitrary initial definition of the parameters, setting the cutoff values to define loss of domain by volumetric methods, without confirmatory studies. However, the usefulness and reliability of its volumetric calculation was later demonstrated by Al-Mansour et al. in a retrospective study with patients who underwent abdominal wall reconstruction between 2007 and 2018.

The description of Sabbagh et al., despite not having been developed with the purpose of predicting loss of domain, brought a useful volumetric calculation that was presented during expert consensus as one of the best descriptions of the volumetric method. Thus, these two methods provide a crucial perspective in preoperative planning, attesting to irreducibility due to lack of intra-abdominal space, the need to use reconstructive techniques to facilitate reduction, and pointing to an increased risk of complications due to increased intra-abdominal pressure.

Sabbagh’s classification had good predictive capacity but with a small population study of 17, and required specific software to carry out the calculations. These were factors that hindered the applicability of the method.

Christy’s component separation index is extremely useful, being almost exclusively dependent on the use of computed tomography and the performance of the predetermined calculation. Although the original study only covered 36 participants, the work by Love et al. ratified its usefulness with a good level of evidence. Despite this, one of the best ways to anticipate the need for relaxation techniques is to use the classification by Love et al., which has become a valuable tool due to its easy application, good predictability, and broad level of scientific evidence attesting to its effectiveness.

Description of computed tomography scans, following the standard report established by Ghandi et al., is beneficial to obtain detailed information about each case. The standard report defined in this study adds information that may be relevant and that may not be contained in other classifications, such as the quality of the abdominal muscles. Another positive aspect of using the standard report is that the checklist for describing the tomographic report highlights the importance of the calculations by Tanaka et al. and Love et al., approaching the two calculations in a complementary way.

The work carried out by the VWHG experts allowed the introduction of new criteria that would be relevant when dealing with potentially complicated hernias. At the time, clinical aspects and the patient’s own risk factors were taken into consideration as a way of predicting unsatisfactory outcomes and, consequently, the complexity of each case. Clinical factors became independent criteria of complexity, without mentioning size or required adjuvant techniques as main issues during this classification. The VHWG work divided groups for OSS risk classification based on clinical factors, patient comorbidities, and the appearance of the lesion during surgical repair. In the work, the risk of OSS was taken as one of the main adverse outcomes, together with the risk of recurrence. The allocation of groups in this classification relied on quality of scientific evidence. However, the study did not have clinical validation to attest to its real clinical usefulness. Kanters et al. subsequently demonstrated the usefulness of the criteria defined by the VHWG in a study with a large sample of patients. During the study, OSS was the main outcome addressed. At the end of the multivariate statistical analyzes, the authors noted that the groups previously defined by the VHWG members should be modified to better adapt to the statistics found in the population study. Considering the need to analyze patients’ clinical factors and the status of subcutaneous cellular tissue, patients with abdominal hernias should be allocated to the Kanters’s classification for OSS predictability and, consequently, case complexity.

The Slatter’s classification does not fit into any of the classifications alone, and the authors’ intention in defining complexity criteria addressed clinical aspects of the patients and descriptive aspects of the hernial defect. However, it is worth highlighting that despite not being a complete and unanimous approach, the complexity classification proposed by Slater et al. can be important for stratifying complex hernias and comes closest to analyzing the main parameters to define an IH as complex. This simultaneous approach provides a comprehensive and informed assessment essential for the successful treatment of complex abdominal wall hernias.

We emphasize that the studies’ eligibility criteria considered better level of scientific evidence, greater relevance in the literature, and updating, and classifications that used redundant methods were only sometimes cited among the references for each topic. The literature review carried out for this study was conducted under predefined inclusion and exclusion criteria, which may have introduced a bias in the selection of identified studies. Furthermore, obtaining gray literature was predominantly carried out through manual review by the authors, resulting in a possible limited view of the field of study. Moreover, the diversity in methodologies of the included studies makes direct comparison and synthesis of data difficult due to disparities in the collection and presentation of information. 

## CONCLUSION

Complex hernias of the abdominal wall can be defined by characteristics analyzed together and which are related to the patient’s previous clinical status, size and location of the hernial defect, status of the subcutaneous cellular tissue, use of preoperative adjuvant techniques, myofascial release techniques, and other complicating factors. In this study, we presented the main classifications that show an adequate level of scientific evidence and listed the main information necessary to define a complex hernia.
